# Shared decision-making experiences regarding urinary diversion among Chinese patients with muscle-invasive bladder cancer: A qualitative study

**DOI:** 10.1016/j.apjon.2026.101024

**Published:** 2026-09-09

**Authors:** Keping Zhu, Ying Zhang, Yusen Wang, Lu Chen, Wenbo Qiao, Meifang Tan, Jiamei Liu, Kun Li, Sijia Hou

**Affiliations:** a Department of Nursing, The First Affiliated Hospital, Zhejiang University School of Medicine, Hangzhou, Zhejiang Province, China; b School of Nursing, Faculty of Medicine, Yangzhou University, Yangzhou, Jiangsu Province, China; c Zhejiang University School of Medicine, Hangzhou, Zhejiang Province, China; d University of Southern California, Los Angeles, CA, USA

**Keywords:** Bladder cancer, Urinary diversion, Shared decision-making, Treatment decision-making, Qualitative research

## Abstract

**Objective:**

To explore the shared decision-making experiences and needs of Chinese patients with muscle-invasive bladder cancer considering urinary diversion.

**Methods:**

A phenomenological qualitative study was conducted at a tertiary hospital in Zhejiang Province, China. Eighteen participants were purposively recruited and completed 22 face-to-face semistructured interviews between May and September 2024. Data were analyzed using Colaizzi's seven-step method.

**Results:**

Four themes were identified: (1) patterns of involvement ranging from active participation to delegated decision-making; (2) reflections on imperfect treatment options; (3) priorities shaped by the tension between survival and quality of life; and (4) unmet information needs. Involvement and preferences were influenced by participants' knowledge, health status, occupation, family relationships, and other people's treatment experiences. Limited consultation time and difficulty understanding medical information constrained participation.

**Conclusions:**

Decisions about urinary diversion were individualized and embedded in clinical, family, and sociocultural contexts. Nurses and other healthcare professionals should provide staged, plain-language information, assess each patient's preferred level of involvement, include family members according to the patient's wishes, and support clarification of values and priorities throughout the perioperative period.

## Introduction

Bladder cancer ranks among the most prevalent malignancies of the urinary system. According to 2020 global cancer statistics, there were approximately 573,000 new cases and 213,000 deaths attributed to bladder cancer, positioning it as the tenth most common malignancy globally.^1^ In China, 2022 estimates projected 92,000 new cases and 43,000 deaths.^2^ Muscle-invasive bladder cancer (MIBC) constitutes approximately 25% of newly diagnosed bladder tumors.^3^ Radical cystectomy (RC) plus urinary diversion and trimodality therapy are recommended as primary curative treatments for non-metastatic MIBC. The primary urinary diversion techniques include orthotopic neobladder (ONB), ileal conduit (IC), and cutaneous ureterostomy (CU).^4^^,^^5^ ONB involves constructing a neobladder from a segment of ileum, allowing patients to void through the urethra without a stoma, thereby preserving body image and a natural voiding pattern, but increasing risks of urinary incontinence and postoperative urinary retention.^6^^,^^7^ IC diverts urine through a stoma using an ileal segment, requiring a permanent appliance, but avoids the risk of urinary incontinence.^6^ CU connects the ureters directly to the skin without bowel manipulation, offering a simpler procedure with shorter operative time.^6^^,^^7^ A recent systematic review and meta-analysis^8^ examining long-term complications (≥ 5 years) following RC found that IC was associated with reduced urinary stone formation and improved global health scores, while ONB was associated with better urinary function and body image perception. However, studies^9^^,^^10^ have failed to demonstrate significant differences in overall quality of life between patients receiving ONB versus other urinary diversion types postoperatively. Given that different urinary diversion techniques carry different risks and benefits, weighing these options is inherently challenging, making decision-making difficult. Furthermore, treatment selection is not solely a clinical determination, but a preference-sensitive decision, and altered urinary function directly affects patients' body image, daily living, and psychosocial well-being. Therefore, patients' preferences and opinions are particularly important in this decision-making process.^11^

Shared decision-making (SDM) encourages healthcare professionals to fully inform patients about treatment options while incorporating patient preferences and values, ultimately aiming for the decision best aligned with individual health needs.^12^ In one study, 57.4% of participants with bladder cancer preferred to participate in treatment decisions.^13^ However, its implementation in clinical practice remains suboptimal. A Chinese study reported that 54.5% lacked relevant information about the three urinary diversion procedures, while 60.7% experienced moderate-to-severe decision regret.^14^ In a patient preference study of MIBC, nearly 20% of patients reported that their physician did not ask about their treatment preferences.^15^ Therefore, there is a need to explore the SDM experiences of patients with MIBC undergoing urinary diversion, so as to better support patients in treatment decision-making and improve decision satisfaction.

Existing qualitative research has contributed substantially to understanding treatment decision-making in this population. Studies have elucidated the decision-making experiences of patients undergoing RC with urinary diversion,^16^ examined gender differences in bladder cancer treatment choices,^17^ identified patient priorities and informational needs during treatment selection consultations,^18^ and investigated factors influencing patient treatment decisions.^19^ These studies have established that patient decision-making is complex, multidimensional, and influenced by clinical, personal, and social factors. Nevertheless, several important knowledge gaps remain. First, the majority of existing qualitative studies were conducted in Western healthcare systems. In China, medical decision-making is shaped by a distinct cultural and familial context. Treatment decisions are often perceived as family matters rather than purely individual choices, with family members frequently participating in or even leading the decision-making process.^20^ Additionally, patients' trust in their physicians may influence how treatment decisions are made and communicated.^21^ Consequently, findings from Western studies may not fully capture the decision-making dynamics in the Chinese clinical context, underscoring the need for research situated in this specific cultural environment. Second, while one recent study has explored SDM in urinary reconstruction among Chinese patients with bladder cancer from both patient and provider perspectives, its primary focus was on identifying barriers and facilitators to SDM rather than on patients' subjective experiences of the decision-making process.^22^ How patients themselves perceive, interpret, and respond to the decision-making process within their cultural and familial contexts remains underexplored. To our knowledge, the specific knowledge gap addressed by the present study is how Chinese patients with MIBC experience SDM regarding urinary diversion, including their involvement patterns, preferences, reflections, and unmet needs. Therefore, this study employs a phenomenological approach to explore the lived experiences and decision-making needs of Chinese patients during the process of selecting a urinary diversion method for radical cystectomy.

## Methods

### Study design

This study employed a phenomenological approach, which is particularly suited for exploring the essential structure of a phenomenon from participants' first-person perspectives. Accordingly, purposive sampling was used to recruit participants who had lived through the decision-making experience; semi-structured interviews were designed to elicit rich, contextualized accounts of participants' subjective experiences; and Colaizzi's seven-step method was applied to support a systematic distillation of the fundamental structure of lived experiences. Reporting followed the Consolidated Criteria for Reporting Qualitative Research (COREQ) guidelines (Supplementary File 1).

### Participants

Eligible participants were recruited in the urology ward of the First Affiliated Hospital of Zhejiang University School of Medicine from May to September 2024. Initial participants were selected purposively to ensure variation in demographic and clinical characteristics, including gender, age, educational level, occupation, choice of treatment, and treatment stage. Inclusion criteria were: (1) pathologically confirmed MIBC; (2) had undergone or were scheduled to undergo radical cystectomy and met guideline-based eligibility criteria for at least one standard urinary diversion technique;^7^ (3) age ≥ 18 years; (4) aware of the diagnosis, able to provide informed consent, and able to participate in an interview. Exclusion criteria included significant communication barriers or cognitive impairment or a psychiatric condition that precluded informed consent or meaningful participation. Eligible patients were identified through electronic health records prior to admission. On the day of admission, we approached the patient for a brief initial conversation. Participants interviewed postoperatively were generally interviewed 3–14 days after surgery, except C17 and C18, who were interviewed face-to-face during follow-up visits approximately two months after surgery. A total of 18 eligible patients were approached, and all agreed to participate and completed the interviews. Due to the well-established epidemiological pattern that MIBC is approximately three to four times more common in males than in females globally,^23^ no eligible female inpatients with MIBC were available during the study period. We therefore telephoned the two most recent female patients with MIBC who had undergone surgery at our institution and been discharged, and invited them to attend interviews at the hospital.

The sample size was dynamically determined following qualitative research principles, with data saturation monitored concurrently during analysis.^24^ In this study, data saturation was confirmed after conducting 20 interviews with 16 participants (four participants were interviewed twice); two additional interviews were conducted with two participants, and no new codes relevant to the study aim were identified, with the coding framework remaining stable. Data saturation was ultimately determined through consensus discussion among the research team.

### Data collection

Data were collected through face-to-face, semi-structured interviews conducted in a quiet and private doctor's office within the urology ward by the Head Urology Nurse (K.Z.) and a doctoral student (Y.Z.), both of whom had received training in qualitative methods and interviewing techniques. After patient admission, interviewers established rapport through preliminary discussions with patients and family members regarding clinical status. The Head Urology Nurse (K.Z.) also participated in participants' clinical nursing care, which further facilitated trust and rapport before the interviews. Written informed consent was obtained from all participants prior to enrollment. During each interview, interviewers observed and documented non-verbal cues in field notes and employed communication strategies to capture patients' authentic experiences. Family members were not present during the interviews to ensure that participants could speak freely without external influence. Interviews were audio-recorded and lasted 20–40 minutes, with an average duration of 33 minutes. The reported duration refers to the actual interview time and does not include the rapport-building period. The semi-structured interview guide was developed specifically for this study, informed by research objectives and expert consultation. Following pilot testing with three convenience-sampled patients (who were not included in the main analysis), the guide was refined by removing redundant questions, optimizing topic sequencing, and improving data collection effectiveness. The final guide is provided as Supplementary File 2. All interviews were conducted jointly by both interviewers (K.Z. and Y.Z.), with K.Z. leading the questioning and Y.Z. operating the audio recorder and taking supplemental field notes.

### Data analysis

Within 24 hours of each interview, two researchers collaboratively reviewed the interview materials. All audio recordings were transcribed verbatim jointly by both researchers (K.Z. and Y.Z.). To ensure accuracy, Y.Z. subsequently verified all transcripts against the corresponding audio recordings. All transcripts were then imported into NVivo 12 for management and coding. For the four participants interviewed at both time points, their preoperative interviews contained limited decision-related content. Postoperative interviews were therefore conducted to further explore and enrich their decision-making experiences. During analysis, the two interviews for each participant were integrated chronologically as a single case. Data analysis followed Colaizzi's seven-step phenomenological method: (1) two researchers (K.Z. and Y.Z.) independently read all transcripts multiple times to gain a holistic understanding of participants' experiences; one researcher (Y.Z.) verified the transcripts against the audio recordings to ensure accuracy; (2) independently extracted significant statements related to surgical options, disease information, personal experiences, and decision-making participation; (3) formulated meanings from these statements through line-by-line coding; (4) compared and clustered the formulated meanings into emergent themes and sub-themes through an iterative process of grouping codes with similar meanings; (5) subcategories were synthesized into broader themes representing key aspects of participants' experiences; (6) developed an exhaustive description of the phenomenon and distilled this description into the fundamental structure of the experience; (7) validated the findings through member checking, whereby summarized results were returned to all 18 participants via telephone or WeChat for accuracy verification. All 18 participants provided confirmatory feedback and no substantive corrections were requested. Their feedback was documented and incorporated to maintain authenticity and avoid researcher influence.

### Rigor

To ensure scientific rigor, all researchers received training in qualitative methods before data collection. Preliminary discussions with patients and their families regarding clinical status, combined with the interviewer's (K.Z.) participation in participants' clinical nursing care, helped establish trust and rapport, enhancing the credibility of the study. However, we also recognized that this dual role could introduce power imbalance and social desirability bias. To minimize the potential bias, we assured participants that their identities would remain anonymous and that their responses would be kept confidential and used solely for research purposes, and that their participation would not affect their clinical care. We also emphasized that there were no right or wrong answers, and encouraged participants to speak freely. Additionally, the presence of both interviewers during each session allowed for mutual monitoring and complementary probing, which further enhanced the trustworthiness of the data collected. Transferability was supported by thick descriptions of participant characteristics, study context, and the phenomenon under investigation. Dependability was ensured via an audit trail documenting the research process, coding decisions, and theme development, along with investigator triangulation—two researchers independently coded all transcripts and resolved discrepancies through team discussion. Confirmability was maintained through reflexivity. Before and during data analysis, we documented our potential assumptions, including expectations that cultural and familial factors might shape patient decision-making, and engaged in regular team discussions to examine how these preconceptions might influence data interpretation. Reflexivity memos were maintained throughout data collection and analysis to record and bracket our preconceptions, ensuring that findings were grounded in participants' accounts rather than our prior assumptions.

## Results

In this study, a total of 18 patients with MIBC were interviewed 22 times. To protect participant confidentiality, all identifiers were removed, and participants were anonymized using codes C1–C18. The participants ranged in age from 50 to 89 years, with a mean age of 67.7 years. The selected urinary diversion types were ileal conduit (*n* = 10), cutaneous ureterostomy (*n* = 6), and orthotopic neobladder (*n* = 2). Regarding interview timing, 4 patients were interviewed only before surgery, 10 patients only after surgery, and 4 patients were interviewed both before and after surgery. Participant characteristics are detailed in Table 1.

**Table 1:** Participant characteristics (*N* = 18).

ID	Sex	Age (years)	Education	Occupation	Marital status	Selected urinary diversion	Interview timing
C1	Male	57	Primary school	Worker	Married	IC	Preoperative + postoperative
C2	Male	86	Primary school	Farmer	Married	CU	Preoperative + postoperative
C3	Male	69	Junior high school	Farmer	Widowed	IC	Preoperative
C4	Male	65	Primary school	Retired	Married	ONB	Preoperative
C5	Male	69	No formal education	Farmer	Married	IC	Postoperative
C6	Male	64	Junior high school	Retired	Married	IC	Preoperative
C7	Male	61	Junior high school	Retired	Married	IC	Preoperative + postoperative
C8	Male	70	Junior high school	Retired	Married	IC	Postoperative
C9	Male	68	Junior high school	Retired	Married	IC	Postoperative
C10	Male	71	Primary school	Retired	Married	IC	Postoperative
C11	Male	60	Junior high school	Retired	Married	CU	Postoperative
C12	Male	70	Junior high school	Retired	Married	IC	Postoperative
C13	Male	70	Junior high school	Retired	Married	IC	Postoperative
C14	Male	50	University	Police officer	Married	ONB	Preoperative + postoperative
C15	Male	64	Junior high school	Retired	Married	Bilateral CU	Preoperative
C16	Male	89	University	Retired	Married	CU	Postoperative
C17	Female	77	Senior high school	Retired	Divorced	CU	Postoperative
C18	Female	58	Senior high school	Worker	Married	CU	Postoperative

“Preoperative” indicates that the patient was interviewed only before surgery; “Postoperative” indicates that interviews were usually 3–14 days after surgery; C17 and C18, interviewed face-to-face at follow-up approximately two months after surgery; “Preoperative + Postoperative” indicates that the patient was interviewed both before and after surgery. ONB, orthotopic neobladder; IC, ileal conduit; CU, cutaneous ureterostomy.

Fig. 1 presents the proposed interpretive framework of the urinary-diversion decision-making experience, inductively derived from participants' accounts. It represents a conceptual synthesis of the key contextual factors and thematic domains that shaped patients' decision-making experiences, rather than a directly observed longitudinal pathway. The decision-making process is embedded within four interconnected contextual domains that collectively shape patients' deliberations. At the core of the framework, a closed cyclical loop visualizes the iterative, interactive nature of the decision-making process, which involves continuous reciprocal interactions among four key stakeholders: patients, healthcare professionals, family members, and peers. Four core themes derived from our qualitative findings are mapped onto the framework, reflecting involvement patterns, treatment reflections, priority trade-offs, and unmet information needs.

**Fig. 1:**
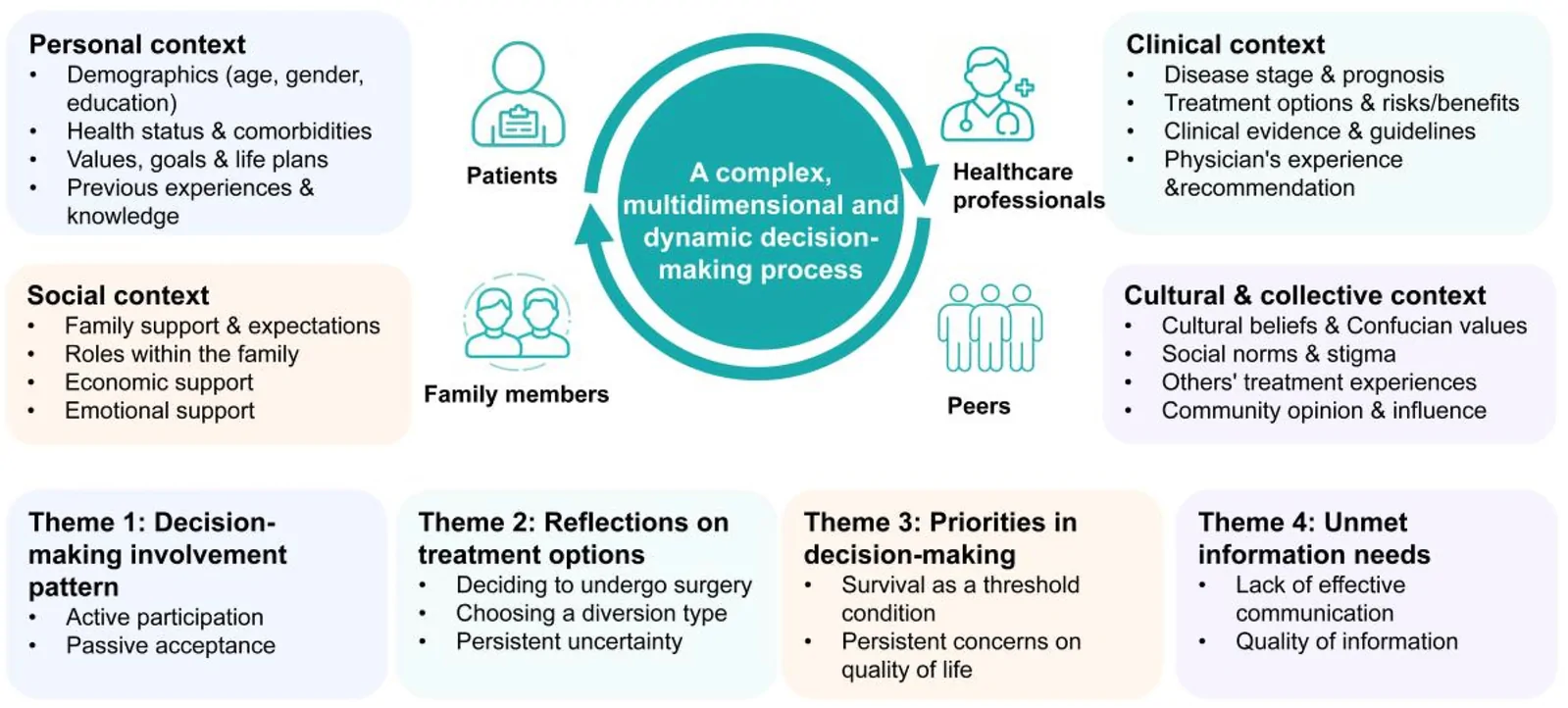
Interpretive framework of urinary diversion decision-making experiences among Chinese patients with muscle-invasive bladder cancer.

### Theme 1: decision-making involvement pattern: from autonomous choice to delegated authority

Treatment decision-making represents a complex, clinically specialized, and highly individualized process for each patient with MIBC. Recommendations from social networks, educational level, and social support shape patients' decision-making involvement patterns.

#### Active participation: patients with prior information assuming decisional responsibility

Patients' decision-making involvement fell along a continuum shaped by preexisting knowledge and perceived decisional self-efficacy. Several patients entered the clinical encounter with preparatory disease information obtained from relatives or friends. This prior exposure to information appeared to reduce their uncertainty about the treatment options and lessen their sole reliance on physicians, which in turn gave them a sense of confidence to participate in the decision-making process and accept decisional responsibility. One participant (C5), whose cousin is a doctor, noted that *“He told me about this disease in detail, so I have some knowledge”*; another (C6), having learned from relatives and friends about potential complications, stated, *“I participated in the preoperative discussion and signature, and I can make the decision myself.”* A third participant (C8) explicitly embraced accountability by declaring, *“Since the illness is on me, I should take the responsibility myself.”* Even when family members attended preoperative discussions, the final decision often rested with the patient. One man (C9) explained that although his son agreed with the doctor's recommendation to remove the bladder, *“If I didn't agree, my family would not proceed with the operation.”* Another (C13), despite limited comprehension, asserted, *“I can make the decision myself, and my wife listens to me.”*

#### Passive acceptance: delegating choice under conditions of uncertainty and trust

Many patients found the medical information too complex to fully comprehend and felt they lacked the expertise to evaluate treatment options. Concerns about making an inappropriate choice or facing future regret further discouraged them from taking an active role. This perceived inability to bear the responsibility of the decision led them to delegate decision-making authority to others. Two distinct patterns emerged. The first was delegation to physicians. Patients placed their trust in physicians' professional expertise and in the reputation of the hospital, believing that doctors were best positioned to determine the most appropriate treatment. For these patients, deferring to physicians was a deliberate strategy to manage the uncertainty of a high-stakes decision. As one participant (C3) claimed, *“We came here because of its reputation, and we trust the doctors. Doctors will have our best interests at heart.”* While another (C7) stated, *“I listen to the doctor … I don't know anything, and following the doctor's advice won't be wrong. I will actively cooperate.”*

The second was delegation to adult children, grounded in family relationships. Patients viewed their adult children as more capable of understanding complex medical information and as trustworthy individuals who would prioritize their well-being. Adult children were also the ones who would be caring for them after surgery, which made their involvement in the decision feel natural. One participant (C4) admitted, *“I don't know about this disease and can't choose by myself. My son thinks it's better not to use a urostomy pouch; I entrusted my son to make the decision.”* Another (C10) described a mediated process: *“The doctor first talks to my daughter, and then she tells me about it. She agreed and signed the consent form. She will take care of me in the future.”* These accounts reveal that passive acceptance is not merely an absence of agency but an adaptive response to knowledge gaps and the perceived burden of decision-making responsibility.

### Theme 2: reflections on treatment options: choosing among imperfect alternatives

Patients' reflections on treatment options unfolded throughout the decision-making process, each layer of thinking shaped by its own concerns. Yet no choice ever felt entirely right; each option came with its own trade-offs, risks, and unknowns. What patients ultimately did was choose the option that seemed least wrong to them, not because it was perfect, but because it was the one they could most accept living with.

#### Deciding to undergo surgery: weighing the risks of surgery against the risks of avoidance

For most patients, the decision-making process began when they were told by their physicians that radical cystectomy was the recommended treatment. This recommendation, however, triggered two distinct patterns of thinking, each revealing how deeply personal concerns shaped their response to the same clinical advice. Some patients, particularly those who perceived themselves as physically frail or burdened by existing health conditions, were consumed by uncertainty about whether surgery would truly be effective and whether their bodies could withstand such a major operation. Their hesitation stemmed from a deep concern that surgery might not deliver the expected benefit, or might even cause more harm than good. One patient (C7), who had a cardiac history, expressed his apprehension: *“My heart isn't in great condition, and I'm a bit worried. Could such a large surgery carry risks? I'm also very concerned about how my body will recover after the operation.”* Another (C5) voiced similar doubts: *“Will such a major surgery go smoothly? I'm worried whether the tumor can be completely removed during the operation, or if it has spread.”* Despite these concerns, they ultimately accepted the surgery.

Other patients, by contrast, reached the same decision through a different line of thinking. Having witnessed close relatives or acquaintances with bladder cancer suffer poor outcomes after choosing conservative treatment or delaying surgery, they became deeply fearful that they might suffer the same fate if they did not act promptly. This fear propelled them to actively choose radical cystectomy as the safer course of action. One participant (C2) recalled: *“A relative of mine had the same disease. He refused to have his bladder removed initially, and by the time the cancer recurred, the doctor said the optimal treatment window had passed, with a poor outcome afterward. I'm grateful that I chose radical cystectomy.”* Another participant (C6), who had witnessed his son-in-law's father experience severe chemotherapy-induced side effects, similarly rejected non-surgical approaches, explaining: *“That would just complicate things—I'd be asking for trouble. The doctor said if the outcome isn't favorable, I'd still need surgery with a urostomy pouch.”*

#### Choosing a diversion type: assessing clinical suitability, pursuing personal priorities

Once patients had committed to surgery, a second layer of decision-making emerged: selecting the specific type of urinary diversion. At this stage, patients engaged in more nuanced trade-offs, weighing factors such as age, health status, occupational demands, and anticipated quality of life against the clinical characteristics of each option.

For older patients or patients with frailty, physicians often recommended CU due to its shorter operative time and lower surgical risk. One older patient (C2) recounted his doctor's reasoning: *“The doctor said that this surgery (CU) is simple and suitable for me. For younger people, they’d need to take out part of their bowel and put it back in—that takes two or three hours longer. I'm too old for that.”* This guidance provided a clear rationale that aligned with his age and health status. For younger and healthier patients, physicians indicated that ONB was an option, as it offered the possibility of preserving natural voiding function and avoiding a permanent stoma. Patients in this group often valued the prospect of maintaining a more natural appearance and voiding pattern, and many believed that their relatively good health would enable them to tolerate the more complex procedure and its potential complications. One younger patient (C4) stated: *“The doctor thought that since I'm still young and have no underlying conditions like hypertension or diabetes, I'm eligible for an ONB.”*

For one participant in physically demanding or image-sensitive occupations, the prospect of a permanent stoma raised concerns about their ability to work or maintain their professional identity. Their decision was driven more by what they wanted to avoid than what they stood to gain, leading them to prioritize options that avoided a stoma. One patient (C14), a police officer in his 50s, explained: *“Put yourself in my shoes—I'm only 50, far from retirement, and I am a police officer; this would leave me disabled.”*

In the SDM process, physicians offered clinical recommendations based on patients' age and health status. Patients, in turn, assessed whether these recommendations aligned with their own conditions and needs. When they found a match, they proceeded to further consider what mattered most to them beyond the clinical basics.

#### Persistent uncertainty: facing an unknowable future

Throughout the decision-making process and long after, patients remained uncertain about what their future life would hold. They struggled to imagine whether things would ever get better, what complications might arise, whether they could manage those complications, and what further treatments might be needed. This uncertainty was not confined to the period before surgery; it persisted through hospitalization, through recovery, and into the return to daily life. The decision was not the conclusion of patients' concerns, but rather the beginning of a longer process of learning to live with its consequences.

One patient (C5) voiced a profound sense of hopelessness: *“There's really no point in choosing among these surgeries. They all feel like bad options. Even if the bladder is completely taken out, I still can't feel at ease—it's too likely to come back. My life might even end up worse than before.”* Another patient (C14), who had been informed that the surgery involved bowel manipulation, asked: *“Could there be intestinal leakage afterward? If a neobladder is created, will urination return to normal in the future? Will sexual function be completely lost?”*

For patients considering or having undergone IC or CU, concerns focused on stoma care, including how to select and change the pouching system, how often it needed to be replaced, and how to prevent complications. One patient (C3) asked: *“How often should I change this pouch? Can it be purchased in the hospital? Is it covered by medical insurance?”* Another patient (C2), five days after surgery, was already worrying about discharge: *“You've managed it well these two days, but what will I do after discharge?”*

### Theme 3: priorities in decision-making: the dynamic tension between survival and quality of life

Despite pervasive uncertainties regarding surgical intervention, patients facing radical cystectomy with permanent urinary diversion described tension between the dual priorities of “prolonging survival” and “maintaining their original quality of life”.

#### Survival as a threshold condition

Almost all participants positioned survival as a non-negotiable priority that fundamentally reordered how they evaluated treatment trade-offs. Rather than weighing survival and quality of life equally, patients applied a hierarchical logic: life must be secured first; only then could other outcomes matter. As one participant (C1) stated, *“First and foremost, I need to preserve my life. Only by surviving can there be hope.”* Another participant (C2) explained, *“As long as I'm alive, everything will be manageable.”* Life is the prerequisite for all future possibilities; postoperative challenges can be coped with only if life itself is preserved. Patients did not deny that urinary diversion would reduce quality of life, but they actively recalibrated this loss as acceptable in exchange for extended survival. For example, one participant (C3) noted, *“A longer life is worth a slight reduction in quality of life”*, while another (C5) remarked, *“Life comes first. Wearing a urostomy pouch is a little ugly, but living a few more years makes it all worthwhile.”* Notably, this survival-first logic did not preclude hopes for bladder preservation: a participant (C5) acknowledged that bladder-sparing options would be preferable, yet when unavailable, radical surgery with diversion became the necessary second-best pathway.

#### Persistent concerns on quality of life despite survival prioritization

Even after electing radical cystectomy with urinary diversion, patients did not cease to worry about daily functioning; rather, these concerns persisted alongside their survival-first logic. One participant (C1) reported, *“I can no longer carry heavy loads, run, hike, or travel far. I feel embarrassed to stay overnight at others' homes.”* While another (C3) described, *“With the pouch, I worry it might protrude when I squat or bend, and that bathing might become troublesome for fear of infection.”* Beyond functional limitations, urinary diversion fundamentally altered body integrity and triggered psychosocial distress. Participants articulated how the stoma restricted mobility, marred appearance, burdened families, and exposed them to perceived stigma, culminating in anxiety, diminished self-esteem, and shame. One participant (C6), after five years of repeated treatments, confessed, *“Honestly, I feel quite anxious. It has been frustrating all these years, and my family has been burdened by me.”* Another (C1) directly linked the stoma to social devaluation: *“It makes me feel ashamed. What will become of me? Will relatives and friends look down on me?”* A third participant (C10) anticipated withdrawing from community life, stating, *“If I have to wear a pouch, I will probably stop going to the card games. I'm afraid of gossip.”* These accounts reveal that the stoma functions not merely as a physical appliance but as a symbolic marker of difference, prompting anticipatory stigma and self-exclusion. The two female participants both reported pronounced concerns about body image disruption. One female participant (C18) articulated this intersection: *“Imagine a woman like me, someone who loves beauty; how can I bear having to wear a pouch? I feel so low I could die. I have no children; who will care for me when I'm old? I love travelling and sampling cuisines; will the surgery rob me of that joy?”*

### Theme 4: unmet information needs: barriers to comprehension and the limits of informal information sources

Information needs persist throughout the decision-making process for patients with MIBC. Time-constrained clinical consultations and medical jargon barriers frequently result in a superficial understanding of treatment options. While fragmented online resources, non-formalized social networks, and peer experiences expand informational access, they simultaneously compromise professional rigor and consistency.

#### Lack of effective communication

Effective physician-patient communication is crucial for building trust and enabling informed decisions, but physician time constraints often reduce preoperative consultations to perfunctory documentation exercises that overwhelm patients while depriving them of genuine comprehension. One patient (C16) explained, *“The doctors are so busy that I can barely see them … Of course, I'll cooperate fully, but I still wish they could spare some time to talk with me”,* while another (C17) lamented, *“The doctor just hurriedly explained the surgery and asked me to sign. I feel our communication was far too insufficient.”* Even when patients are informed of surgical necessity, the inherent complexity of medical terminology still restricts their understanding of treatment details. One participant (C3) acknowledged, *“I'm not very clear about the exact procedure. I know it's cancer and that I have to wear a urostomy pouch afterwards.”* and another (C14) confessed, *“The doctor talked to me, but I could barely understand.”* Furthermore, significant barriers to communication efficiency persist, as verbal explanations prove insufficient for conveying complex medical concepts. One participant (C18) said, *“I know nothing about this disease. When asked to make a choice, the doctor only talked to me for barely 10 minutes before asking me to sign. I was stunned.”* Another (C17) proposed a practical solution, asking, *“Could there be something like a single sheet, perhaps a table, that lists the pros and cons of each option, along with what daily life will entail afterward? That would make everything clear at a glance.”*

#### Quality of information

High-quality treatment decisions hinge on access to accurate information covering disease fundamentals, surgical details, and postoperative stoma care. However, while patients acquire basic knowledge through traditional and digital media, fragmented online resources compromise medical precision. One participant who searched Baidu reported, *“It said the bladder can't be saved and I'll have to urinate through a pouch on my abdomen.”* Another, though educated and accustomed to reading newspapers, WeChat, and TikTok for nutrition tips, noted that, *“I'd never heard of urinary diversion.”* Beyond online information, patients also consult healthcare professionals within their social networks, comparing recommendations from attending physicians with those from medically trained relatives. One patient (C5) explained, *“My cousin is a doctor, and he explained this disease to me in detail, so I have some understanding,”* while another (C9) stated, *“I have a doctor relative. He told me about this disease, and what he said was the same as Director Shen—no choice but to remove the bladder. Gradually, I accepted it.”* Most influentially, the treatment experiences and long-term outcomes of friends or relatives who have undergone radical cystectomy constitute a pivotal information source. One participant (C5) observed, *“Several villagers have had bladder cancer and wear a pouch; they've been doing well for over ten years.”* Another (C13) visited a neighbour who had the same operation more than five years prior, and recounted, *“He urged me to do the surgery, saying one gets used to it and life remains largely unchanged. With good living conditions and health insurance nowadays, we won't burden our children.”* These narratives demonstrate that while peer experiences provide compelling, concrete reassurance, they also reveal the absence of formal, standardized information channels, leaving patients to rely on fragmented, occasionally inconsistent sources that may introduce bias alongside practical guidance.

## Discussion

### Main findings

This study provides a comprehensive understanding of how Chinese patients with MIBC navigate complex treatment choices for urinary diversion within their clinical and cultural context. Our findings reveal distinct patterns of decision-making involvement and clarify the underlying reasoning that shapes patients' reflections on treatment options, their priorities between survival and quality of life, and their unmet information needs. These insights offer an essential foundation for developing culturally sensitive strategies to support SDM in oncology care.

This study identified two distinct patterns of decision-making involvement among Chinese patients with MIBC: active participation and passive delegation. A subset of patients who had obtained preparatory information from relatives or friends demonstrated greater confidence in engaging with treatment decisions and assuming decisional responsibility, consistent with previous research showing that prior knowledge may reduce uncertainty and support participation.^25^

One form was delegation to physicians. In contrast to Western studies on bladder cancer treatment decision-making, where most patients preferred an active role in decision-making with their providers, delegation to physicians was a prominent pattern in our study.^18^ This difference may be explained by cultural and systemic factors unique to the Chinese context.^22^ When facing high-stakes treatment decisions, patients in our study often recognized that they lacked the medical knowledge to independently evaluate complex treatment options and therefore needed to rely on someone with greater expertise. Physicians, by virtue of their professional training and clinical experience, led patients to trust that they could guide them toward the most appropriate decisions. Moreover, some patients in our sample came from rural areas or lower-level healthcare settings, where specialist expertise was less accessible, and they had deliberately sought care at our tertiary hospital precisely to obtain authoritative medical guidance, a choice that itself reflected their confidence in physician-led decision-making.

The other form was delegation to adult children. Influenced by Confucian familism, major health decisions are often perceived as collective family matters rather than purely individual choices.^20^ In the Chinese clinical context, family members often make treatment decisions alongside or even on behalf of patients, especially in severe illness like cancer. The cultural value of filial piety positions adult children as responsible for the care of aging parents, and as those who would be caring for them after surgery, which further rendered their involvement in the decision natural and expected. Moreover, adult children were often perceived by patients as better educated and better equipped to comprehend medical information and navigate the healthcare system.^26^ Collectively, these factors made patients more willing to entrust decision-making to their adult children. While Western studies have similarly documented family involvement in treatment decision-making,^17^ such delegation to adult children may be more readily accepted and culturally legitimized within the Chinese clinical context, but it should always occur according to the patient's wishes and with the patient's autonomy preserved.

Patients recognized that every available alternative carried significant drawbacks, leading them to actively seek the option they could most accept living with. The initial decision regarding whether to undergo radical cystectomy was influenced by prior exposure to others' treatment experiences. Those who had witnessed poor outcomes from conservative treatment tended to perceive inaction as more dangerous than surgery itself, while those without such experiences focused more on surgical risks. This pattern aligns with social learning theory.^27^ A systematic review of cancer help-seeking in low- and middle-income countries similarly found that exposure to others' illness experiences influences health-related decisions.^28^ Regarding diversion type selection, patients first considered physicians' recommendations and their own health status, then weighed personal priorities, reflecting a progressive process of deliberation. Future decision aids could explicitly incorporate this logic by first clarifying medical appropriateness and then facilitating exploration of personal values, thereby better supporting patients in navigating these complex choices. The persistence of uncertainty across all stages further suggests that decision-making extends beyond the initial choice into postoperative life, indicating that ongoing support should be provided rather than concentrated at the decision point alone.

This study identifies two paramount priorities in patient decision-making: survival and quality of life, consistent with prior research.^18^^,^^19^ Survival often functioned as a threshold condition, as patients viewed life itself as a prerequisite for all future possibilities. Only once survival was perceived as secured did quality-of-life concerns re-emerge and persist, indicating that the tension between these priorities is not resolved by the decision itself. Patients' quality-of-life concerns centered on practical stoma care issues, including pouch management, leakage prevention, and daily self-care, which influenced their postoperative adaptation. Previous research on general ostomy populations has shown that wearing a pouch can fundamentally alter perceived role, self-esteem, body image, sexuality, and social relations.^29^ The daily realities of stoma care shaped how patients anticipated their postoperative quality of life. Occupational identity further shaped these priorities. For patients in physically demanding or image-sensitive professions, the practical and symbolic implications of a stoma were perceived as difficult to reconcile with their professional identity and work demands. This concern strongly influenced their treatment choices and could, in some cases, lead patients to prioritize occupational compatibility. The two female participants in this study expressed prominent concerns about body image, but the data are not sufficient to establish a broader gender-related pattern. Future studies could explore gender-specific needs in decision-making.

Some observational studies report better HRQoL in selected domains after ONB than after IC or CU;^30^^,^^31^ however, nonrandomized designs, selection differences, and heterogeneous measures limit causal comparison. Patients tended to believe that an ostomy would substantially alter body image and amplify psychological burden. Similarly, although observational data in selected older or high-risk populations suggest that CU may offer certain perioperative benefits over IC, such as shorter operative time, shorter hospital stay, less blood loss, and fewer perioperative complications, CU is associated with trade-offs including the potential need for long-term stenting, risks of stomal stenosis, and potential upper-tract infection, with no significant differences in survival or overall quality of life reported between the two procedures.^32^, ^33^, ^34^ Despite these considerations, most patients in our study were unaware of these nuances.

This knowledge gap may contribute to suboptimal decision-making and subsequent regret. Therefore, healthcare professionals should provide accurate, evidence-based information, make an effort to address misconceptions and support balanced understanding, help clarify their personal priorities, and assist them in making high-quality treatment decisions.

This study highlights unmet informational needs as a critical barrier to patient engagement in treatment decision-making for MIBC. Effective physician-patient communication is bidirectional, yet our findings reveal that patients' reflections on treatment options are often limited by insufficient information transmission. Information is mostly conveyed verbally using complex terminology, which exceeds patient comprehension. Additionally, patients struggle to absorb information within limited time and perceive insufficient opportunity to ask questions, contributing to their lack of participation.^35^ This may be attributed to the fact that the information is novel to patients but routine for healthcare professionals, who have repeated it countless times; divergent educational and occupational backgrounds further widen the comprehension gap.^35^ Previous studies^36^^,^^37^ confirm that SDM requires patients to possess adequate disease knowledge and fully grasp the information provided. Therefore, healthcare professionals should convey information in plain language, maintain honesty, and allocate sufficient time for assimilation and clarification. A patient decision aid for urinary diversion has been developed and found acceptable to patients and clinicians.^38^ Therefore, healthcare professionals should identify which information is relevant to patients, provide such information at the appropriate time, and avoid overwhelming patients with excessive information at once, which may prevent them from capturing the information they need.^35^

### Implications for nursing practice and research

#### Providing structured preoperative education and informed consent

Preoperative education should provide structured information covering each diversion type, including surgical procedures, postoperative recovery, stoma care, anticipated quality of life, and long-term self-management.^39^ The use of visual materials and teach-back methods is recommended to enhance comprehension and confirm patient understanding. Decision aids in uro-oncology may improve knowledge and risk perception; evidence specific to urinary diversion remains limited.^12^

#### Addressing sociocultural factors in communication and decision support

Nurses should be aware of key sociocultural factors when communicating with patients and families, including patients' trust in physician authority, Confucian familism, filial piety, and patients' prior exposure to others' treatment experiences. Recognizing these factors can help identify patients' decision-making styles and improve the effectiveness of preoperative communication. When families are actively involved in decision-making, healthcare providers should extend communication from the individual patient to the family unit, respecting patient autonomy while acknowledging the family's role in information sharing and decision negotiation. For patients who highly delegate decision-making authority to physicians or adult children, providers should help clarify their key information needs and ensure that their core concerns are addressed, rather than having them passively receive information.^14^

We also suggest that decision aids be designed with sociocultural factors in mind, respecting each patient's preferences for autonomy and family involvement, and providing information and options that are aligned with the Chinese social and cultural context, thereby better supporting patients in understanding their choices, clarifying personal priorities, and facilitating effective communication among patients, families, and clinicians.

#### Clarifying decision priorities and values

Healthcare providers should help patients articulate their values regarding survival, body image, daily functioning, work, and caregiver burden, and integrate these concerns into clinical discussions to help align treatment decisions with patients' life circumstances and reduce the risk of decision regret.^40^ A continuous care pathway spanning preoperative counseling, postoperative support, and long-term follow-up can help patients navigate their evolving priorities across the treatment trajectory. Future research could further explore how these decision priorities change over time and incorporate these findings into the design of continuous care models to optimize patient-centered decision support.

### Limitations

This study has several limitations. First, participants were interviewed at different time points relative to surgery (before surgery, after surgery, or both), which may have influenced their accounts of decision-making, information needs, and decision regret. Second, the inclusion of only two female patients risks overlooking gender-specific decision-making experiences and perspectives. Third, the exclusive focus on patient perspectives without integrating insights from family caregivers or healthcare professionals limits a comprehensive understanding of SDM challenges in clinical practice. Finally, participants were recruited solely from a single tertiary-care hospital in China; variations across healthcare systems and cultural values in other countries or ethnic groups may limit the generalizability of our findings. Future studies should incorporate broader sociodemographic variables to elucidate their relationships with the decision-making process among radical cystectomy patients.

## Conclusions

This qualitative study explores the decision-making experiences of Chinese patients with MIBC undergoing urinary diversion surgery, identifying four key themes: (1) decision-making involvement pattern; (2) reflections on treatment options; (3) priorities in decision-making; and (4) unmet information needs. Patients' treatment decisions are influenced by age, education level, occupation, health status, and peer-shared experiences. Unmet information needs emerged as a barrier to patient engagement in decision-making. Variations emerged in patient preferences and priorities, with most prioritizing survival while a subgroup emphasized quality of life during decision-making. To support informed, preference-concordant decisions, we recommend that healthcare professionals deliver personalized information support tailored to patients' characteristics and fully understand patients' preferences and priorities. Additionally, the use of patient decision aids and encouraging patients to express their opinions can be beneficial in the decision-making process.

## CRediT authorship contribution statement

**Keping Zhu:** Conceptualization, Methodology, Writing - original draft, Data curation, Formal analysis. **Ying Zhang:** Methodology, Writing - original draft, Data curation, Formal analysis. **Yusen Wang:** Methodology, Formal analysis, Writing - review & editing. **Lu Chen:** Methodology, Formal analysis, Investigation, Writing - review & editing. **Wenbo Qiao:** Methodology, Writing - review & editing. **Meifang Tan:** Writing - review & editing. **Jiamei Liu:** Writing - review & editing. **Kun Li:** Writing - review & editing. **Sijia Hou:** Writing - review & editing. All authors read and approved the manuscript.

## Ethics statement

Ethical approval was granted by the Clinical Research Ethics Committee of the First Affiliated Hospital, College of Medicine, Zhejiang University (Approval No. 2024-0432). In accordance with the Declaration of Helsinki, written informed consent was obtained from all participants before their involvement.

## Data availability statement

The data supporting the findings of this study are available from the corresponding author, KZ, upon reasonable request.

## Declaration of Generative AI and AI-assisted technologies in the writing process

During the preparation of this work, the authors used DeepSeek for translation and language editing. After using this tool, the authors reviewed and edited the content as needed and take full responsibility for the content of the article.

## Funding

This work was supported by Zhejiang Provincial Medical and Health Technology Project (Grant No. 2024KY1005) and the Discipline Construction Project Fund for Nursing Research, FAHZU (Grant No. 2025ZYHL28). The funders had no role in the study design, data collection and analysis, decision to publish, or preparation of the manuscript.

## Declaration of competing interest

The authors declare no conflict of interest.
