# Supplementary material for: Shared decision-making experiences regarding urinary diversion among Chinese patients with muscle-invasive bladder cancer: A qualitative study
Source: Asia Pac J Oncol Nurs. 2026 Sep 9;13:101024. doi: 10.1016/j.apjon.2026.101024 (PMC13635918; doi:10.1016/j.apjon.2026.101024)
Supplement: Supplementary file 1 [file mmc1.docx]

**COREQ checklist.**

| **COREQ CHECKLIST** | **Reported in Manuscript** |
| --- | --- |
| **Domain 1: Research team and reflexivity** |  |
| *Personal Characteristics* |  |
| 1. Interviewer/facilitator: Which author/s conducted the interview or focus group? | Yes. Line 140-141, Page 5 |
| 2. Credentials: What were the researcher’s credentials? | Yes. Line 143-145, Page 5 |
| 3. Occupation: What was their occupation at the time of the study? Registered Nurse/Midwife | Yes. Registered Nurse |
| 4. Gender: Was the researcher male or female? | Yes. Female |
| 5. Experience and training: What experience or training did the researcher have? | Yes. Line 143-145,188-189, Page 5 and 7 |
| *Relationship with participants* |  |
| 6. Relationship established: Was a relationship established prior to study commencement?  Yes, not a formal relationship, some participants may have known the names of, and met some of the research team over the course of their careers. | Yes. Line 141-145,189-197 Page 5 and 7 |
| 7. Participant knowledge of the interviewer: What did the participants know about the researcher? | Head Urology Nurse: Yes ,Line 141-143, Page 5  Ph.D. candidate: No |
| 8. Interviewer characteristics: What characteristics were reported about the interviewer/facilitator? | Yes. Line 143-145,188-189, Page 5 and 7 |
| **Domain 2: Study design** |  |
| *Theoretical framework* |  |
| 9. Methodological orientation and Theory: What methodological orientation was stated to underpin the study? | Yes. Phenomenological approach  Line 98-105, Page 4 |
| *Participant selection* |  |
| 10. Sampling: How were participants selected? | Yes. Line 100,110, Page 4 |
| 11. Method of approach: How were participants approached? | Yes. Line 118-123, Page 4-5 |
| 12. Sample size: How many participants were in the study? | Yes. 18 patients.Line 123-124, Page 5 |
| 13. Non-participation: How many people refused to participate or dropped out? Reasons? | No,all 18 eligible patients approached agreed to participate.Line 123-124, Page 5 |
| *Setting* |  |
| 14. Setting of data collection: Where was the data collected? | Yes. Line 138-159, Page 5 and 6 |
| 15. Presence of non-participants: Was anyone else present besides the participants and researchers? | No, no one else was present.Line 138-140, Page 5 |
| 16. Description of sample: What are the important characteristics of the sample? | Yes. Line 214-221, Table 1,Page 8 |
| *Data collection* |  |
| 17. Interview guide: Were questions, prompts, guides provided by the authors? Was it pilot tested? | Yes. Line 152-157, Page 6 |
| 18. Repeat interviews: Were repeat interviews carried out? If yes, how many? | Yes, four patients carried out two interviews, others only one.Line 165-167, Page 6 |
| 19. Audio/visual recording: Did the research use audio or visual recording to collect the data? | Yes, audio.Line 150, Page 5 |
| 20. Field notes: Were field notes made during and/or after the interview or focus group? | Yes. Line 146-148, Page 5 |
| 21. Duration: What was the duration of the interviews or focus group? | Yes. Line 150, Page 5 |
| 22. Data saturation: Was data saturation discussed? | Yes. Line 130-136, Page 5 |
| 23. Transcripts returned: Were transcripts returned to participants for comment and/or correction? | No |
| **Domain 3: Analysis and findings** |  |
| *Data analysis* |  |
| 24. Number of data coders: How many data coders coded the data? | Yes. Line 169-173, Page 6 |
| 25. Description of the coding tree: Did authors provide a description of the coding tree? | No.The manuscript describes the analytic process and presents a conceptual framework, but does not provide a coding tree. |
| 26. Derivation of themes: Were themes identified in advance or derived from the data? | Derived from the data.Line 169-186, Page 6-7 |
| 27. Software: What software, if applicable, was used to manage the data? | Yes. Nvivo 12. Line 164-165, Page 6 |
| 28. Participant checking: Did participants provide feedback on the findings? | Yes. Line 181-186, Page 7 |
| *Reporting* |  |
| 29. Quotations presented: Were participant quotations presented to illustrate the themes/findings? Was each quotation identified? e.g. participant number | Yes.Page 10-18 |
| 30. Data and findings consistent: Was there consistency between the data presented and the findings? | Yes. Page 10-18 |
| 31. Clarity of major themes: Were major themes clearly presented in the findings? | Yes. Page 10-18 |
| 32. Clarity of minor themes: Is there a description of diverse cases or discussion of minor themes? | Yes. Page 10-18 |
