# Supplementary material for: Shared decision-making experiences regarding urinary diversion among Chinese patients with muscle-invasive bladder cancer: A qualitative study
Source: Asia Pac J Oncol Nurs. 2026 Sep 9;13:101024. doi: 10.1016/j.apjon.2026.101024 (PMC13635918; doi:10.1016/j.apjon.2026.101024)
Supplement: Supplementary file 2 [file mmc2.docx]

**Semi-structured Interview Guide for Bladder Cancer Patients**

**Introduction to Key Concepts**

We are interested in learning about your experience in making decisions regarding surgery for bladder cancer, particularly about the process of choosing a type of urinary diversion. Your insights will help us improve communication and support for future patients.

**Interview Questions**

1. Could you describe your understanding of bladder cancer and the different types of urinary diversion? How did you learn about this information?
2. Thinking back to your conversations with your surgical team, what information was most helpful to you?
3. What were the most important factors that influenced your choice of surgery? Was there any specific information that helped you make your final decision?
4. In an ideal situation, how would you have liked to be involved in making decisions about your surgery? What kind of support would have made this process easier for you?
5. Did you face any difficulties or challenges when trying to make a decision? If so, what were they?

**Conclusion**

6. Is there anything else you would like to share about your experience or any suggestions you have for improving how patients are supported in making these types of decisions?

Thank you very much for your time and for sharing your experience. Your feedback is very important and will help us improve care for future bladder cancer patients.
